# Synovium‐On‐A‐Chip: Simulating the Microenvironment of the Rheumatoid Arthritis Synovium via Multicell Interactions to Target Fibroblast‐Like Synoviocytes

**DOI:** 10.1002/advs.202511945

**Published:** 2025-09-23

**Authors:** Wenya Diao, Yi Jiao, Tingting Deng, Jienan Gu, Bailiang Wang, Qidong Zhang, Peilong Wang, Ning Xu, Cheng Xiao

**Affiliations:** ^1^ China–Japan Friendship Clinical Medical College Beijing University of Chinese Medicine Beijing 100029 China; ^2^ Institute of Clinical Medical Sciences China–Japan Friendship Hospital Beijing 100029 China; ^3^ Department of Orthopedics China–Japan Friendship Hospital Beijing 100029 China; ^4^ Institute of Quality Standard and Testing Technology for Agro–Products Chinese Academy of Agricultural Sciences Beijing 100081 China

**Keywords:** fibroblast‐like synoviocytes, microfluidics, organ‐on‐a‐chip, rheumatoid arthritis, synovium

## Abstract

Fibroblast‐like synoviocytes (FLSs) play pivotal roles in the synovial inflammation of rheumatoid arthritis (RA) and are promising therapeutic targets. Currently, few in vitro models effectively mimic the characteristics of RA FLSs. In this study, microfluidic chips, 3D culture systems, and flow‐based culture techniques are integrated to develop a vascularized and immune‐activated synovium‐on‐a‐chip (SOC) model. Using immunofluorescence staining, multiparametric flow cytometry, and ELISA, the optimal coculture ratio of RA FLSs, M1‐type macrophages, and human umbilical vein endothelial cells is determined to be 1:1:1 (2 × 10^6^ cells/mL). Analyses of inflammatory cytokine profiles from 22 blood samples, 48 synovial fluid samples, and six synovial tissues demonstrate that the SOC model consistently maintains elevated levels of interleukin (IL)‐6 and IL‐8 over 9 days, closely recapitulating RA synovial inflammation. Additionally, RA FLSs in the SOC model exhibit elevated levels of Cadherin‐11, matrix metalloproteinase (MMP)‐1, MMP‐3, and Ki‐67. By evaluating the therapeutic efficacy and toxicity of triptolide and celastrol, this study confirms the potential of the SOC model in predicting in vivo drug responses, thereby offering a promising tool for preclinical drug assessment in RA‐related research.

## Graphical abstract

The synovium‐on‐a‐chip (SOC) model establishes a 3D flow‐based co‐culture of rheumatoid arthritis (RA) fibroblast‐like synoviocytes (FLSs) with M1 macrophages and endothelial cells on a microfluidic chip. This model better recapitulates the inflammatory microenvironment of RA synovium and the invasive/proliferative properties of RA FLSs in vivo, offering a platform for mechanistic studies and drug screening targeting FLSs.

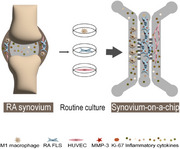

## 1 Introduction

Rheumatoid arthritis (RA) is a systemic autoimmune disease characterized by persistent synovial inflammation and progressive joint destruction,^[^ ^1^ ^]^ affecting ≈1% of the global population.^[^ ^2^ ^]^ Synovitis develops rapidly in the early stages of the disease and persists throughout its progression, significantly contributing to the poor prognosis of RA.^[^ ^3^ ^]^ Despite significant advancements in treatment options such as disease‐modifying antirheumatic drugs, biologics, and Janus kinase inhibitors over the past two decades, challenges remain, including low rates of sustained remission of synovitis and treatment failure in some patients.^[^ ^4^ ^]^ Fibroblast‐like synoviocytes (FLSs), critical components of synovial tissue, play pivotal roles in the development of RA synovitis and exhibit tumor‐like proliferation and invasive characteristics under pathological conditions.^[^ ^5^, ^6^ ^]^ FLSs not only display pathogenic features within the inflammatory microenvironment but also actively drive the progression of synovitis.^[^ ^7^ ^]^ Targeting FLSs represents a promising therapeutic strategy for RA.^[^ ^8^ ^]^ Research on RA FLSs models may provide novel insights into potential treatment options for RA.^[^ ^9^ ^]^ Therefore, constructing an in vitro synovial model capable of accurately reproducing the pathological state of RA FLSs is of paramount importance. In vivo, synovitis arises from the complex interactions of multiple cell types, and the function of RA FLSs is modulated by other cells, such as M1‐type (M1) macrophages and endothelial cells. Myeloid‐derived macrophages accumulate in the RA synovium and differentiate into M1 macrophages, which produce proinflammatory cytokines such as tumor necrosis factor‐alpha (TNF‐α), interleukin (IL)‐6, and IL‐1β, thereby exacerbating synovitis.^[^ ^10^, ^11^ ^]^ Crosstalk between FLSs and macrophages may represent a critical mechanism underlying synovitis.^[^ ^12^, ^13^ ^]^ Synovial vessels are essential for the expansion of synovial tissue and the development of RA synovitis.^[^ ^14^, ^15^, ^16^ ^]^

Compared with inflammatory models established using proinflammatory cytokines (e.g., IL‐1β and TNF‐α), multicellular inflammatory models more closely recapitulate the inflamed RA synovium. However, establishing these types of models remains a formidable challenge. Traditional 2D cultures and Transwell cocultures cannot be used to establish complex in vitro models that faithfully replicate physiological conditions. In contrast, 3D tissue in vitro models, which incorporate an extracellular matrix, better mimic the in vivo state than 2D cultures.^[^ ^17^ ^]^ Previous studies have employed 3D cocultures of FLSs with macrophages and endothelial cells to simulate the RA synovium in vitro.^[^ ^18^, ^19^, ^20^ ^]^ Into establish these coculture models, the three cell types are mixed within a culture dish, making the construction of an in vitro vascular network or the precise targeting of RA FLSs difficult. Organ‐on‐a‐chip technology integrates multicell separation and coculture with microfluidic techniques on a microscale, enabling the precise spatiotemporal control of cell culture,^[^ ^21^ ^]^ construction of in vitro vascular networks,^[^ ^22^ ^]^ simulation of immune cell perfusion and migration,^[^ ^23^ ^]^ replication of core tissue and organ functions (e.g., digestion or fluid movement),^[^ ^24^ ^]^ and dynamic multidimensional drug screening.^[^ ^25^ ^]^ Microfluidic organ‐on‐a‐chip technology provides unique advantages in recreating physiological and pathological microenvironments^[^ ^26^, ^27^ ^]^ and enables real‐time high‐resolution imaging as well as dynamic analysis of cellular, biochemical, and metabolic activities.^[^ ^28^ ^]^ In previous RA microfluidic synovial models, researchers have investigated single‐cultured FLSs to construct 3D synovial tissue,^[^ ^29^ ^]^ cocultures of FLSs with chondrocytes to model the synovium‐cartilage axis,^[^ ^30^ ^]^ and cocultures of FLSs with human umbilical vein endothelial cells (HUVECs) to develop vascularized synovial chip models.^[^ ^31^ ^]^ Despite these advancements, a critical gap remains in the availability of microfluidic synovial chip models that are capable of accurately mimicking RA FLSs within the RA synovial microenvironment.

Here, we developed a 3D flow coculture system of RA FLSs with M1 macrophages and HUVECs on a microfluidic chip to construct a vascularized and immunologically relevant synovial chip model for the precise targeting of RA FLSs. We systematically optimized the type and concentration of the 3D culture matrix, the density of each cell type, and the ratio of the three cell types to more accurately reproduce the RA synovial microenvironment in vitro. These optimizations not only enhanced the standardization and reproducibility of our model but also improved its ability to reflect the inflammatory state, invasiveness, and proliferative capacity of RA synovial tissue under physiological conditions. Furthermore, we conducted preliminary evaluations of the efficacy and cytotoxicity of triptolide (TP) and celastrol (CEL) in this model, demonstrating its potential as a platform for studying drugs specifically targeting FLSs for the treatment of RA.

## 2 Results

### 2.1 Generation of the Synovium‐On‐A‐Chip Model with 3D Coculture of RA FLSs, M1 Macrophages, and HUVECs Under Medium Perfusion

**Figure 1:**
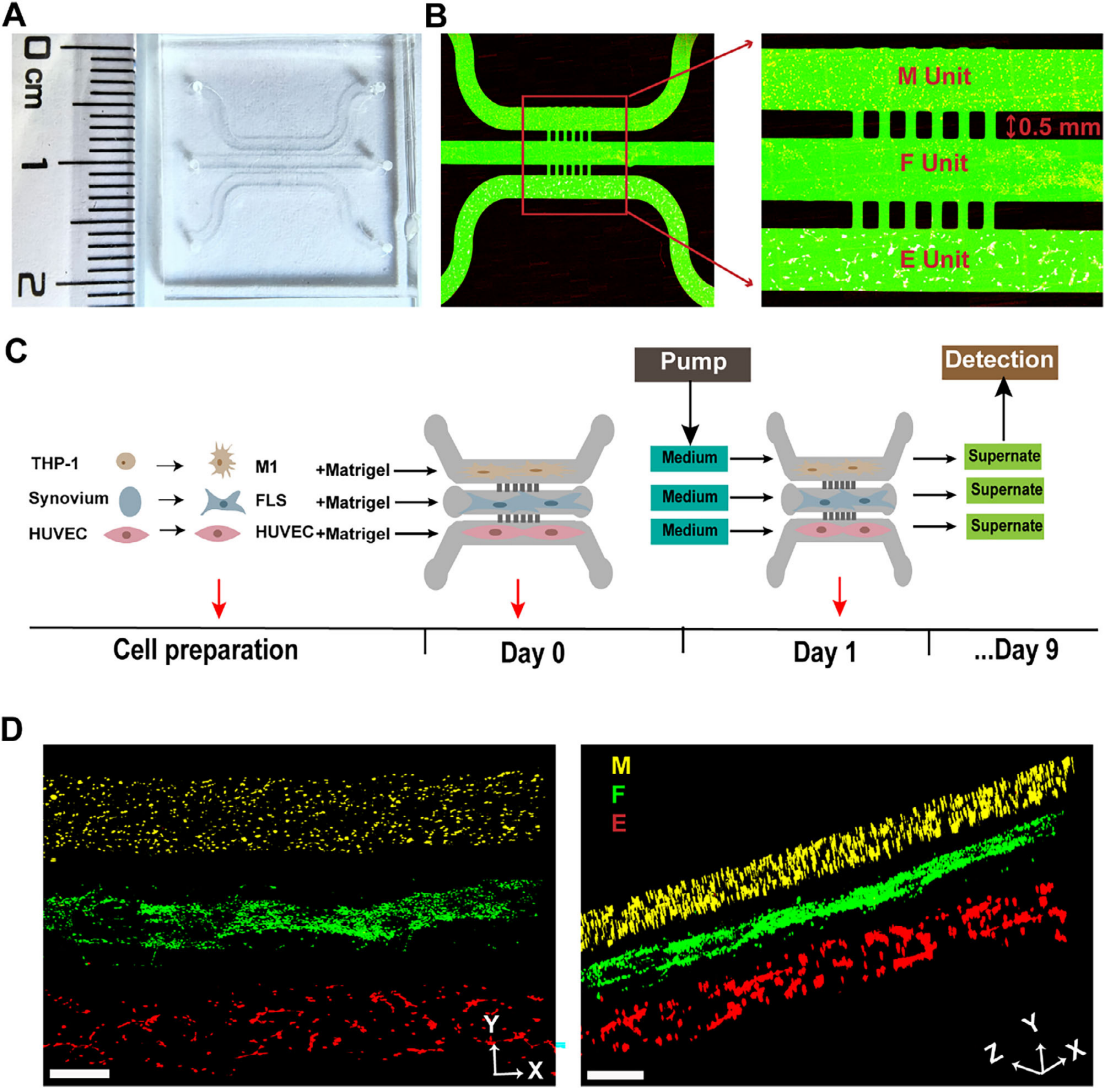
Construction of the rheumatoid arthritis (RA) synovium‐on‐a‐chip model. A) Physical image of the synovial chip. B) Confocal microscopy images of the channels of the synovial chip, with the middle channel being the fibroblast‐like synoviocytes (FLSs) channel (F Unit) and the two side channels being the M1‐type (M1) macrophages channel (M Unit) and the human umbilical vein endothelial cells (HUVECs) channel (E Unit). C) Schematic diagram of the culture method used for the synovial chip. On day 0, FLSs extracted from synovial tissue were inoculated into the middle channel, M1 macrophages induced from THP‐1 cells were inoculated into the upper channel, and untreated HUVECs were inoculated into the lower channel. Beginning on day 1, flow culture was initiated using a peristaltic pump at a rate of 3 µL h^−1^. D) Confocal microscopy images obtained during 3D coculture (yellow = M1 macrophages, green = RA FLSs, red = HUVECs). Scale bars: 500 µm.

A three‐channel microfluidic chip was utilized to construct the synovium‐on‐a‐chip model (**Figure** **1**A). Specifically, RA FLSs (F Unit), which were isolated from the synovial tissue of RA patients and characterized as Vimentin^+^CD68^−^ by flow cytometry (Figure S1A, Supporting Information), were positioned in the central channel, whereas M1 macrophages (M Unit) and HUVECs (E Unit) were seeded in the lateral channels (Figure 1B). M1 macrophages were induced from THP‐1 monocytes and confirmed as inducible nitric oxide synthase (iNOS)^+^CD68^+^ by immunofluorescence (IF) staining (Figure S1B, Supporting Information). HUVECs were sourced from a commercially available cell line. IF staining confirmed the expression of intercellular adhesion molecule‐1 (ICAM‐1) (an indicator related to the activation of synovial endothelial cells in rheumatoid arthritis) in HUVECs under 3D culture conditions (Figure S1C, Supporting Information).

The interconnection among the three cell types was established through directional fluid flow at a controlled rate of 3 µL h^−1^, which was generated by a peristaltic pump coupled to the chip inlet. Under this flow condition, cells maintain proper 3D morphology and physiological function. The resulting microchannel shear stress (∼7.8 dyn/cm^2^) falls well within the safe range for most cell types. The effluent from the outlet was subsequently collected and subjected to detection and analysis (Figure 1C). We assessed the viability of the three cell types in Matrigel, type I rat tail collagen, gelatin methacryloyl, and fibronectin using live/dead staining to identify the optimal matrix for the 3D growth of the three cell types. The results showed that all three cell types exhibited high viability when cultured in Matrigel (Figure S1D, Supporting Information). Consequently, Matrigel was selected as the culture matrix. Based on prior studies,^[^ ^32^ ^]^ we further optimized the concentration of Matrigel, a critical parameter that dictates pore size and consequently influences its permeability. Following FITC‐dextran perfusion, the permeability of Matrigel was found to be most appropriate at a Matrigel concentration of 25% (Figure S1E, Supporting Information). The bright‐field microscopy images revealed that the morphology of RA FLSs remained largely unchanged before and after flow culture at this concentration (Figure S1F, Supporting Information). Under these conditions, RA FLSs, M1 macrophages, and HUVECs were successfully cocultured in 3D (Figure 1D).

### 2.2 Optimization of the Seeding Densities for Each of the Three Cell Types in the Synovium‐On‐A‐Chip Model

We first optimized the seeding densities of the three cell types to sustain proper cell growth and functional activity on the chip for a more accurate simulation of the inflammatory microenvironment of the RA synovium. Four seeding densities, 0.5 × 10^6^ cells/mL, 1 × 10^6^ cells/mL, 2 × 10^6^ cells/mL, and 4 × 10^6^ cells/mL, were used for each of the three cell types. We labeled RA FLSs with Vimentin (a fibroblast marker) and Cadherin‐11 (CDH11) (a functional indicator reflecting the invasive and proliferative characteristics of RA FLSs), and found that the relative fluorescence intensity of CDH11 in RA FLSs was highest at an inoculation density of 2 × 10^6^ cells/mL. M1 macrophages were labeled with CD68 (a macrophage marker) and iNOS (a functional indicator of M1 macrophages polarization), and the results of IF staining showed that the expression of iNOS in M1 macrophages peaked at 2 × 10^6^ cells/mL.

**Figure 2:**
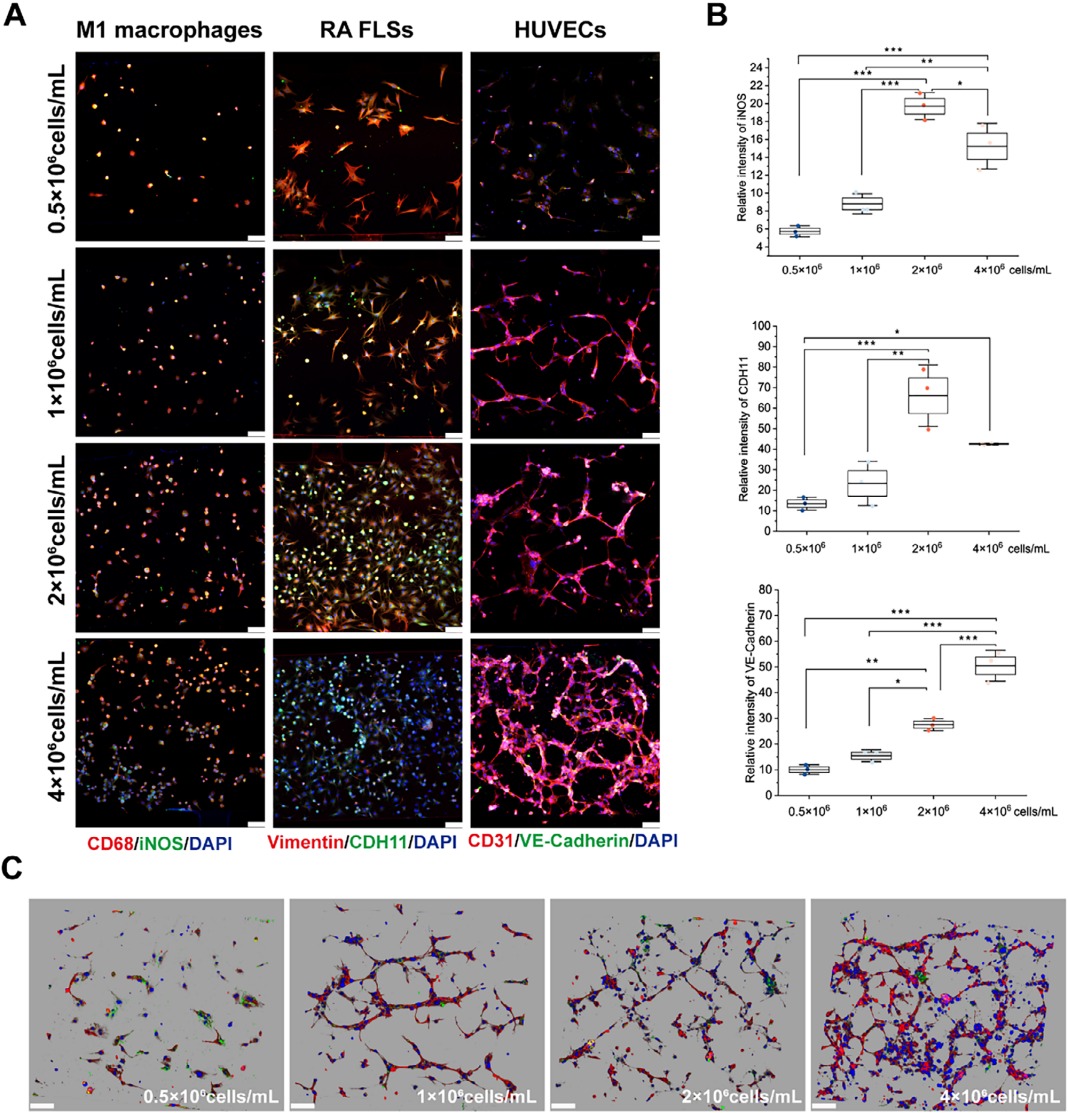
Density‐dependent characteristics of M1 macrophages, RA FLSs, and HUVECs. A) Confocal microscopy images of M1 macrophages, RA FLSs, and HUVECs cultured at various densities. Scale bars: 100 µm. B) Quantitative analysis of the relative fluorescence intensity for protein expression in the three cell types at different densities. M1 macrophages express inducible nitric oxide synthase (iNOS), RA FLSs expressing Cadherin‐11 (CDH11), and HUVECs express VE‐Cadherin. C) 3D images of HUVECs structures cultured under varying density conditions. Scale bars: 100 µm. *n* = 3 per group. Data are presented as the mean ± SD. Statistical analysis: one‐way analysis of variance (ANOVA) with Tukey's test. *p < 0.05, **p < 0.01, and ***p < 0.001.

CD31 (an endothelial cell marker) and VE‐Cadherin (a key adhesion molecule for preserving endothelial stability) were used to label HUVECs. Unlike that of RA FLSs and M1 macrophage, the relative fluorescence intensity of VE‐Cadherin progressively increased with increasing cell density (**Figure** **2**A,B). Furthermore, 3D imaging of HUVECs at different seeding densities revealed that cells cultured at 0.5 × 10^6^ cells/mL failed to form vascular structures, whereas those at 1 × 10^6^ cells/mL or higher successfully formed vascular networks. Notably, the lumen diameter of the formed vessels was inversely correlated with increasing cell density (Figure 2C). Based on these results, we selected a seeding density of 2 × 10^6^ cells/mL for the culture of RA FLSs and M1 macrophages on the chip, and the density of HUVECs was further determined through the experiments described below.

### 2.3 Optimization of Seeding Ratios for the Three Cell Types Through an Assessment of Inflammatory Features in the Synovium‐On‐A‐Chip Model

**Figure 3:**
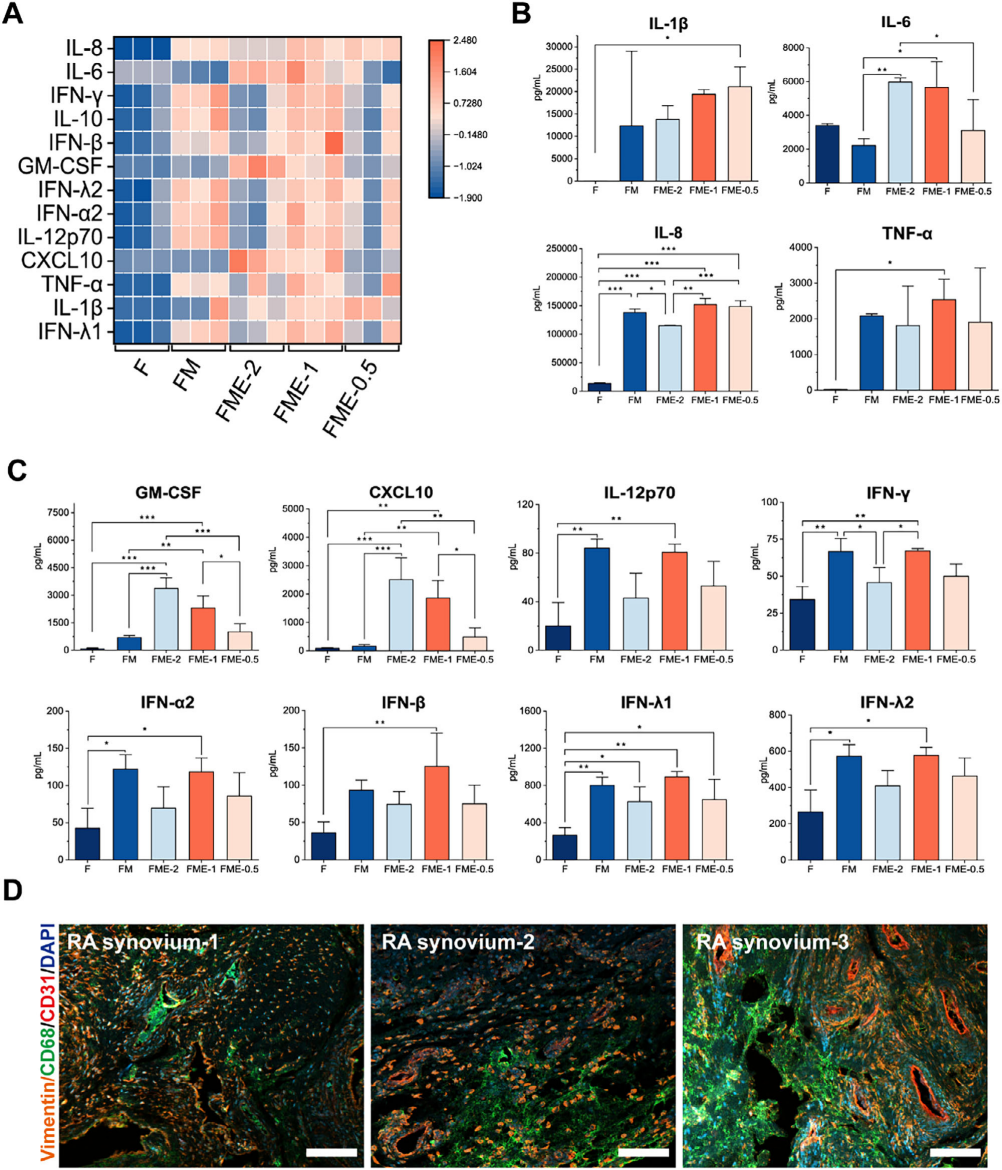
Expression levels of inflammatory factors in cocultures of RA FLSs, M1 macrophages, and HUVECs at different seeding ratios. A) Heatmap illustrating the expression levels of multiple inflammation‐related factors secreted by RA FLSs cultured alone (F), RA FLSs cocultured with M1 macrophages (FM), and RA FLSs, M1 macrophages, and HUVECs cocultured at ratios of 1:1:2 (FME‐2), 1:1:1 (FME‐1), and 1:1:0.5 (FME‐0.5). B) Expression levels of interleukin (IL)‐1β, IL‐6, IL‐8, and tumor necrosis factor‐alpha (TNF‐α) in the F, FM, FME‐2, FME‐1, and FME‐0.5 groups. C) Expression levels of granulocyte macrophage‐colony stimulating factor (GM‐CSF), C‐X‐C motif chemokine ligand 10 (CXCL10), IL‐12p70, interferon (IFN)‐γ, IFN‐α, IFN‐β, IFN‐λ1, and IFN‐λ2 in the F, FM, FME‐2, FME‐1, and FME‐0.5 groups. D) Multiplex immunofluorescence staining for specific proteins, including Vimentin (mesenchymal marker), CD68 (macrophage marker), and CD31 (endothelial cell marker), in RA synovial tissue. Scale bars, 100 µm. *n* = 3 per group. Data are presented as the mean ± SD. Statistical analysis: one‐way ANOVA with Tukey's test. *p < 0.05, **p < 0.01, and ***p < 0.001.

We assessed the inflammatory status of five groups including single RA FLSs culture (F), RA FLSs and M1 macrophages coculture at a 1:1 ratio (FM), and three‐cell cocultures of RA FLSs, M1 macrophages, and HUVECs at ratios of 1:1:2 (FME‐2), 1:1:1 (FME‐1), and 1:1:0.5 (FME‐0.5) by measuring the levels of multiple inflammatory cytokines on day 1 to further determine the optimal seeding ratio of HUVECs with a fixed FLSs/M1 macrophages ratio of 1:1. The results showed that the FME coculture presented significantly higher levels of proinflammatory cytokines than the FM and F cocultures (**Figure** **3**A). Specifically, among the cytokines closely associated with RA pathogenesis (IL‐6, IL‐8, IL‐1β, and TNF‐α),^[^ ^33^ ^]^ the IL‐6 expression levels in the FME‐2 and FME‐1 cocultures were higher than those in the FM coculture (*p* < 0.05) (Figure 3B). However, the IL‐8 expression level in the FME‐2 coculture was lower than that in the FM coculture (*p* < 0.05) (Figure 3B). The expression levels of other inflammatory cytokines, such as granulocyte macrophage‐colony stimulating factor (GM‐CSF) and C‐X‐C motif chemokine ligand 10 (CXCL10), were higher in the FME‐2 and FME‐1 cocultures than in the FM coculture (*p* < 0.05) (Figure 3C). Similar to the IL‐8 levels, interferon (IFN)‐γ expression in the FME‐2 coculture was also lower than that in the FM coculture (*p* < 0.05) (Figure 3C).

We further determined the ratios of the three cell types in the synovium by performing multiplex immunohistochemical (mIHC) staining on synovial sections from RA patients, using Vimentin (for RA FLSs), CD68 (for macrophages), and CD31 (for endothelial cells) as markers to estimate the relative abundances of these three cell types in the inflamed synovium. The analysis revealed that the number of endothelial cells was lower than that of FLSs and macrophages (Figure 3D). In addition, we conducted immunohistochemical (IHC) and tissue IF staining assays on synovial tissues using CD68 and CD34 (an endothelial progenitor cell marker) to exclude the potential influence of nonspecific antibody staining. The results revealed that the 3,3′‐diaminobenzidine (DAB) staining intensity of CD68 and CD34 in the synovial tissues of RA patients was significantly higher than that in those of osteoarthritis (OA) patients (Figure S2A, Supporting Information), whereas the expression levels of these two markers were partially consistent in the RA sample (Figure S2B, Supporting Information). Taking all the above results into account, we adopted a cell ratio of 1:1:1 in subsequent experiments.

### 2.4 Investigating the Inflammatory Cytokine Dynamics of the Synovium‐On‐A‐Chip Model

**Figure 4:**
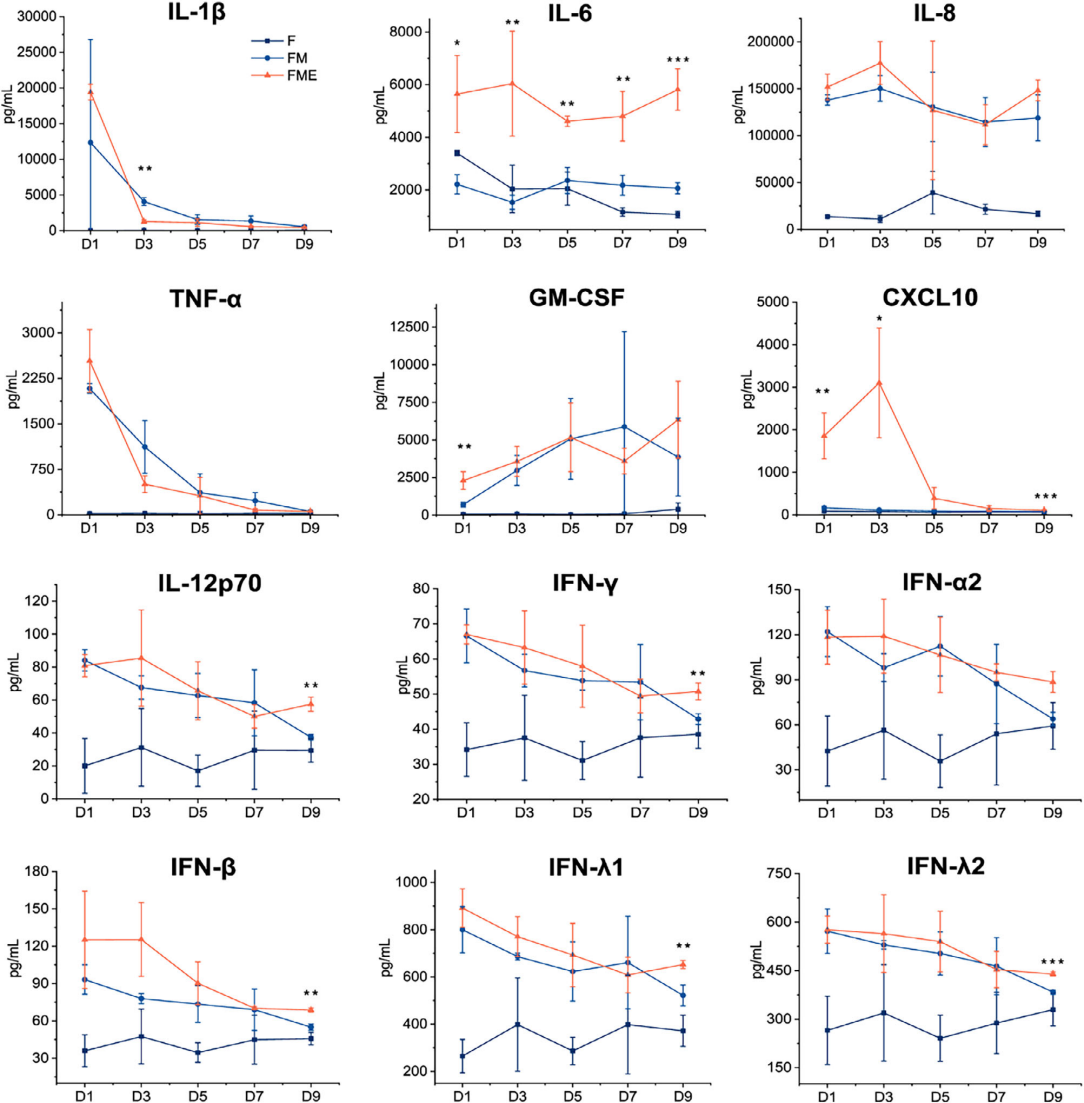
Expression levels of inflammatory factors (IL‐1β, IL‐6, IL‐8, TNF‐α, GM‐CSF, CXCL10, IL‐12p70, IFN‐γ, IFN‐α2, IFN‐β, IFN‐λ1, and IFN‐λ2) on different days (days 1–9) in the F, FM, and FME (FME‐1) groups. The data are presented as the mean ± SD. *n* = 3 per group. Data are presented as the mean ± SD. Statistical analysis: independent samples *t*‐test for the FME group compared with the FM group. *p < 0.05, **p < 0.01, and ***p < 0.001.

We conducted a detailed evaluation of inflammatory factor expression from day 1 to day 9 to investigate the dynamic expression levels of inflammatory factors in the model. Compared with the FM and F groups, the FME group maintained significantly higher levels of inflammatory factors over the entire 9‐day period in the absence of any exogenous stimulation (**Figure** **4**). Specifically, on day 9, the expression levels of IL‐6, CXCL10, IL‐12p70, IFN‐γ, IFN‐β, IFN‐λ1, and IFN‐λ2 in the FME group remained significantly higher than those in the FM group (*p* < 0.05). Notably, the fluctuations in IL‐6 and IL‐8 levels in the FME group were relatively stable throughout the 9‐day period. The sustained high expression levels of IL‐6 and IL‐8 over the 9‐day observation period further indicated that the functional states of these three cell types remained relatively stable throughout the experimental duration. Compared with that in the FM group, the expression level of IL‐6 in the FME group was consistently elevated over the entire 9‐day period (*p* < 0.05), whereas that of CXCL10 was significantly increased only during the first 3 days (*p* < 0.05).

Notably, the levels of GM‐CSF in the FM and FME groups remained consistently higher than in the F group over 9 days. Accumulating evidence indicates that GM‐CSF plays a critical role in maintaining the M1 phenotype of macrophages,^[^ ^34^ ^]^ suggesting that the co‐culture of RA FLSs with M1 macrophages contributes to the preservation of this phenotypic state. We further analyzed inflammatory factor expression from day 1 to day 9 in the FME‐2 and FME‐0.5 groups to exclude the possibility that the dynamic expression patterns of inflammatory factors were influenced by the number of endothelial cells. The results showed that the trends in inflammatory factor expression in these two groups were comparable to those observed in the FME‐1 group (Figure S3, Supporting Information). Collectively, these findings indicate that our model can sustain a high level of inflammation for an extended period and has distinct advantages in terms of the expression levels of IL‐6, IL‐8, and CXCL10.

### 2.5 Expression Levels of Inflammatory Factors in Clinical Samples from RA and OA Patients

**Figure 5:**
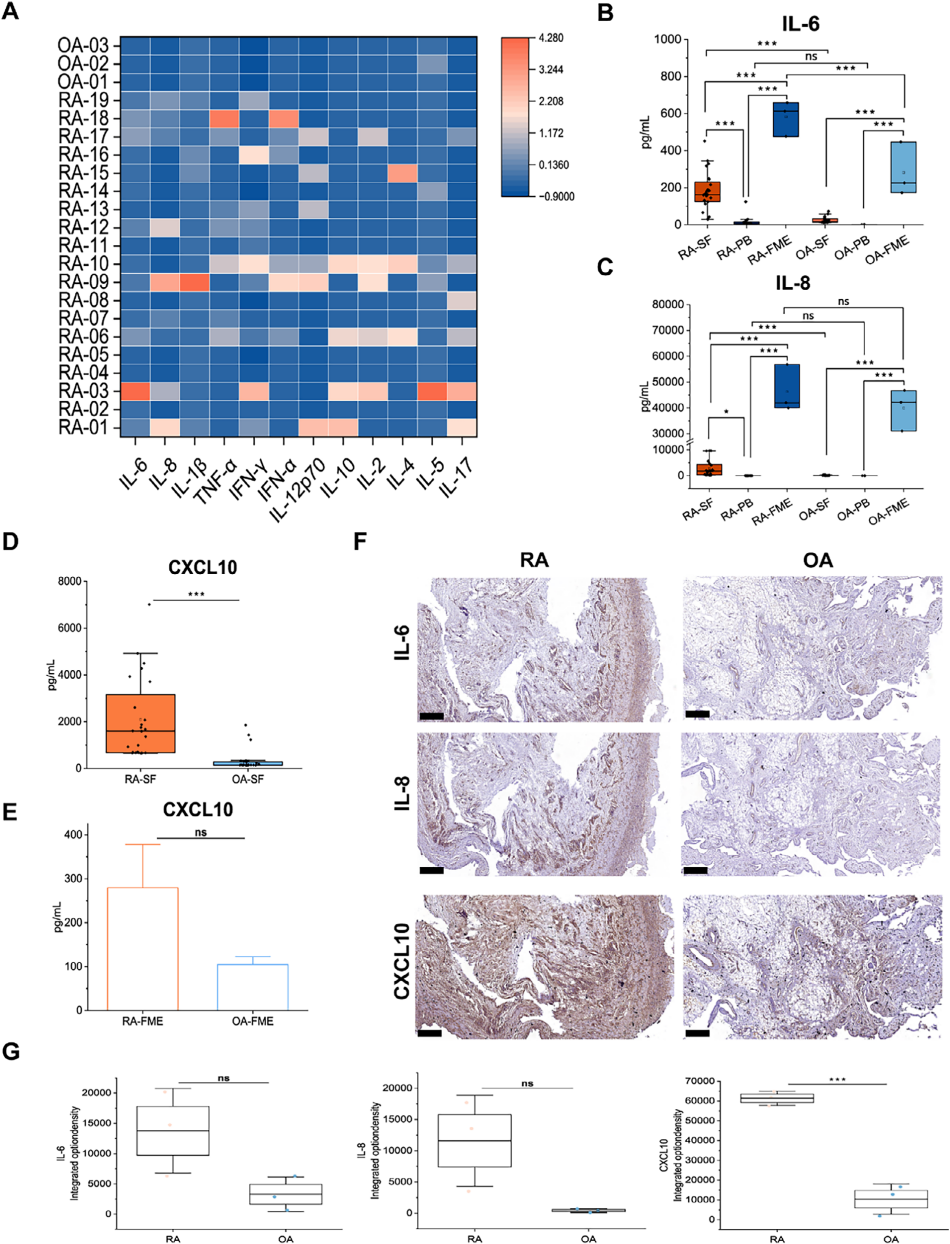
Expression levels of inflammatory factors in clinical samples from RA patients and osteoarthritis (OA) patients. A) Heatmap illustrating the expression levels of multiple inflammation‐related factors in the peripheral blood (PB) of RA and OA patients. B) Quantitative analysis of IL‐6 expression in paired RA/OA‐synovial fluid (SF) (*n* = 24), RA/OA‐PB (*n* = 19, 3), RA/OA‐FME (*n* = 3), RA/OA‐FME from RA/OA 01–03, RA‐PB from RA 01‐19, OA‐PB from OA 01‐03, RA/OA‐SF from RA/OA 01–24. C) Quantitative analysis of IL‐8 expression in paired RA/OA‐SF (*n* = 24), RA/OA‐PB (*n* = 19, 3), RA/OA‐FME (*n* = 3), RA/OA‐FME from RA/OA 01–03, RA‐PB from RA 01‐19, OA‐PB from OA 01‐03, RA/OA‐SF from RA/OA 01–24. D) Quantitative analysis of CXCL10 expression in SF samples from RA and OA patients. *n* = 24. E) Quantitative analysis of CXCL10 expression in FME samples from RA and OA patients. *n* = 3. F) Representative images of immunohistochemical staining for IL‐6, IL‐8, and CXCL10 in synovial tissues from RA and OA patients. Scale bars: 500 µm. G) Graphical representation of the integrated optical density data for IL‐6, IL‐8, and CXCL10 expression in synovial tissues from RA and OA patients. *n* = 3. Data are presented as the mean ± SD. Statistical analysis: independent‐samples *t*‐test for two groups and one‐way ANOVA with Tukey's test for multiple groups. ns, not significant, *p < 0.05, **p < 0.01, and ***p < 0.001.

We analyzed clinical samples, including peripheral blood (PB), synovial fluid (SF), and synovial tissues, from RA and OA patients to evaluate whether the persistent increases in the levels of inflammatory factors (IL‐6, IL‐8, and CXCL10) in our model correspond to the clinical characteristics of the RA synovium. Detailed patient information is summarized in Table S1 (Supporting Information). Among the 22 patients, only a subset of RA patients presented significantly elevated levels of IL‐6, IL‐8, IL‐1β, TNF‐α, and IFN‐α in the PB (**Figure** **5**A). We further compared the expression levels of IL‐6 and IL‐8 in the PB, SF, and FME between the RA and OA groups. To minimize inter‐individual variability, we employed paired PB, SF, and synovial tissue collected from the same patients during the same time window to measure the expression levels of IL‐6 and IL‐8 (RA/OA‐FME from RA/OA 01–03, RA‐PB from RA 01‐19, OA‐PB from OA 01‐03, RA/OA‐SF from RA/OA 01–24). The results revealed that the IL‐6 and IL‐8 levels were markedly higher in the RA‐SF than in RA‐PB or OA‐SF (*p* < 0.05). Notably, the RA‐FME constructed in our study presented even higher levels of IL‐6 and IL‐8 than the RA‐SF (Figure 5B,C).

Additionally, CXCL10 was highly expressed in RA‐SF (*p* < 0.05) (Figure 5D). In coculture experiments involving M1 macrophages and HUVECs, the levels of IL‐6, IL‐8, and CXCL10 were increased in the RA‐FME group compared with the OA‐FME group, with significant differences in the levels of IL‐6 (Figure 5B,C,E). These findings indicate that primary cells derived from RA and OA patients exhibit distinct disease‐specific phenotypes even in the presence of M1 macrophages and endothelial cells. IHC staining of RA and OA synovial tissues confirmed that IL‐6, IL‐8, and CXCL10 were more highly expressed in RA synovial tissues than in OA tissues, with CXCL10 showing the most pronounced difference (Figure 5F,G). Furthermore, we validated the high expression of CXCL10 in the synovium of RA animal models, including collagen‐induced arthritis (CIA) rats and K/BxN mice (Figure S4A, Supporting Information). Findings from the analyses of clinical samples further confirmed that our model has strong clinical relevance and, to a significant extent, accurately recapitulates the pathological characteristics of RA.

### 2.6 Analysis of the Invasive Capacity and Proliferative Properties of RA FLSs in the Synovium‐On‐A‐Chip Model

**Figure 6:**
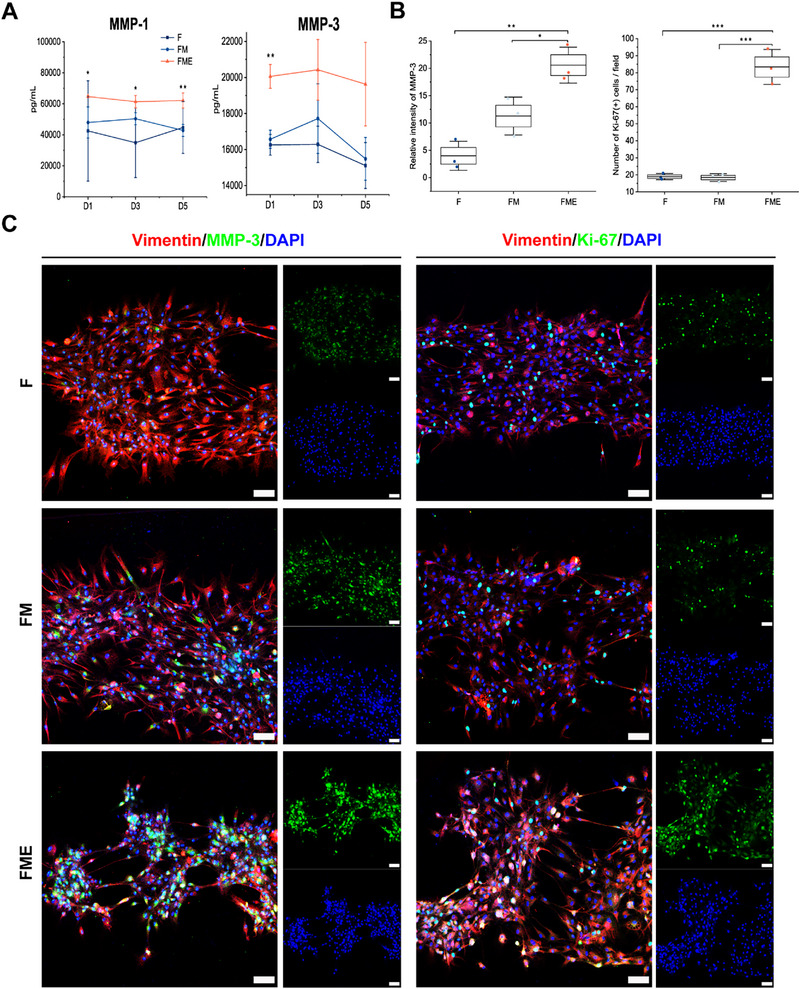
Invasiveness and proliferation of RA FLSs in the F, FM, and FME groups. A) Expression levels of matrix metalloproteinase (MMP)‐1 and MMP‐3 in the F, FM, and FME groups. Independent‐samples t‐test for the FME group compared with the FM group. B) Relative fluorescence intensity levels of MMP‐3 and Ki‐67 expressed by RA FLSs in the F, FM, and FME groups. C) Confocal microscopy images of MMP‐3 and Ki‐67 expression in RA FLSs from the F, FM, and FME groups. Scale bars, 100 µm. *n* = 3 per group. Data are presented as the mean ± SD. Statistical analysis: one‐way ANOVA with Tukey's test for multiple groups, unless otherwise specified. *p < 0.05, **p < 0.01, and ***p < 0.001.

We initially assessed the levels of matrix metalloproteinase (MMP)‐1 and matrix MMP‐3 in the F, FM, and FME groups using ELISA to further elucidate the effects of the other two cell types on the invasive capacity and proliferative characteristics of RA FLSs in the model. The results demonstrated that the levels of MMP‐1 and MMP‐3 were significantly higher in the FME group than in the FM group (*p* < 0.05) (**Figure** **6**A). We subsequently validated the expression of MMP‐3 in RA FLSs under different culture conditions via IF staining. The findings showed a gradual increase in MMP‐3 expression in RA FLSs across the F, FM, and FME groups, with the FME group exhibiting a more pronounced increase than the F and FM groups (Figure 6B,C). The DAB staining intensity of MMP‐3 in the RA synovium was significantly higher than that in the OA synovium (Figure S4B, Supporting Information). These observations indicate that both M1 macrophages and endothelial cells contribute to enhancing the invasiveness of RA FLSs.

Additionally, we detected the expression of Ki‐67 in FLSs to assess their proliferative capacity. The results revealed no significant difference in Ki‐67 expression between the F and FM groups; however, Ki‐67 expression in the FME group markedly increased compared with that in both the F and FM groups (Figure 6B,C). These findings suggest that the presence of endothelial cells significantly enhances the proliferative characteristics of RA FLSs. Collectively, these findings highlight the critical importance of both M1 macrophages and endothelial cells in promoting the invasive capacity and proliferative properties of RA FLSs in vitro.

### 2.7 Application of the Synovium‐On‐A‐Chip Model for Evaluating Drug Efficacy and Toxicity

**Figure 7:**
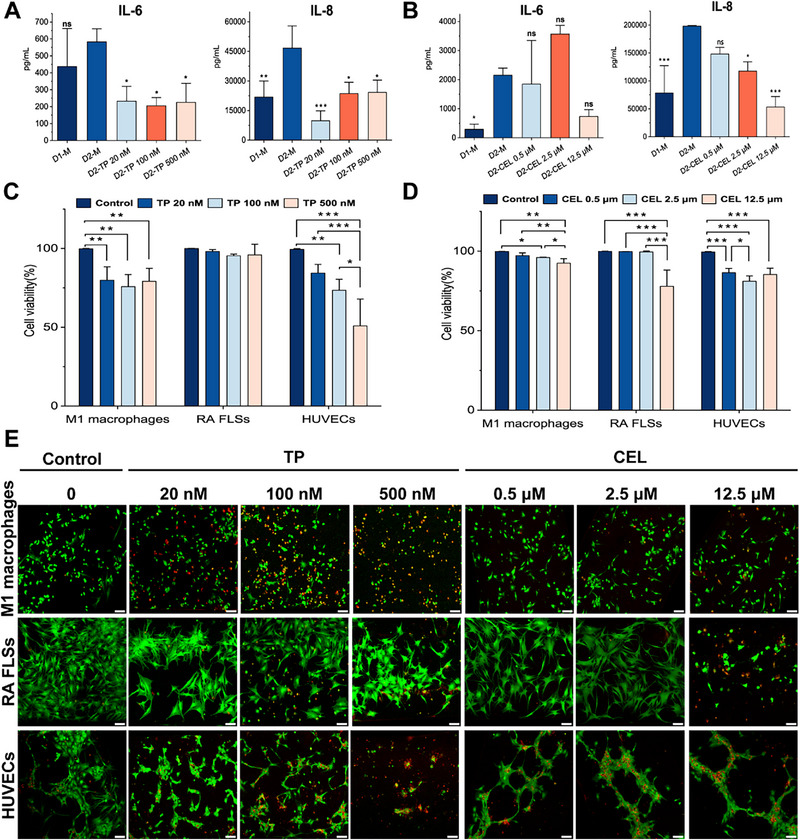
Evaluation of the efficacy and toxicity of triptolide (TP) and celastrol (CEL) in the SOC model. A) Expression levels of IL‐6 and IL‐8 before and after TP administration. One‐way ANOVA with Dunnett's test for multiple comparisons to D2‐M group. B) Expression levels of IL‐6 and IL‐8 before and after CEL administration. One‐way ANOVA with Dunnett's test for multiple comparisons to D2‐M group. C) Survival rates of M1 macrophages, RA FLSs, and HUVECs after TP administration. D) Survival rates of M1 macrophages, RA FLSs, and HUVECs after CEL administration. E) Live and dead staining images of M1 macrophages, RA FLSs, and HUVECs after TP and CEL administration (green = live cells, red = dead cells). Scale bars, 100 µm. *n* = 3 per group. Data are presented as the mean ± SD. Statistical analysis: one‐way ANOVA with Tukey's test, unless otherwise specified. *p < 0.05, **p < 0.01, and ***p < 0.001.

We performed efficacy and toxicity assessments using TP and CEL to further evaluate the applicability of our model for drug research. Based on our previous study on TP^[^ ^35^ ^]^ and published studies on CEL,^[^ ^36^ ^]^ we selected TP at a concentration of 20 nm and CEL at 0.5 µm as the initial drug concentrations. Due to the design of the microfluidic chip, a spatial concentration gradient forms between the central and lateral channels, resulting in lower actual drug concentrations than the initial input. To comprehensively evaluate potential cytotoxicity on M1 macrophages (M unit) and HUVECs (E unit) while covering a wider therapeutic range, we performed experimental assessments using five‐fold serial increments of the initial concentrations. On day 1, TP (20, 100, or 500 nm) or CEL (0.5, 2.5, or 12.5 µm) was added to the F unit, while normal culture medium was introduced into the M unit and E unit. On day 2, the expression levels of inflammatory factors in the model were quantified by ELISA, and cell viability was assessed via live/dead staining. The ELISA results indicated that, compared with the untreated group on day 2 (D2‐M), TP at concentrations of 20, 100, and 500 nm significantly reduced the expression levels of IL‐6 and IL‐8 (*p* < 0.05) (**Figure** **7**A). Additionally, CEL at concentrations of 2.5 and 12.5 µm significantly decreased the expression level of IL‐8 in the model (*p* < 0.05) (Figure 7B). Live/dead staining revealed that triptolide had no significant effect on the survival rate of RA FLSs but markedly reduced the survival rates of M1 macrophages and HUVECs (*p* < 0.05) (Figure 7C,E). All three concentrations of CEL significantly decreased the survival rates of RA FLSs, M1 macrophages, and HUVECs (*p* < 0.05) (Figure 7D,E).

Furthermore, we exposed three types of cells in 2D culture to the drugs at the specified concentrations and supplemented the conditioned medium (2D‐CM) from RA FLSs cultures to M1 macrophages and HUVECs. Thereafter, we assessed the survival rates of the cells under these experimental conditions. The results revealed that all three concentrations of TP significantly reduced the survival rates of M1 macrophages and HUVECs (Figure S5A,B, Supporting Information). Moreover, a high concentration of CEL markedly decreased the survival rates of all three cell types (Figure S5C,D, Supporting Information). Collectively, these findings not only corroborate the observations derived from our model but also validate its applicability for evaluating drug efficacy and toxicity. More importantly, by administering drugs to a single cell type, our model displays the ability to assess the survival of the other two cell types, thereby underscoring its significant potential in drug toxicity evaluation.

## 3 Discussion

RA is characterized by synovial inflammation and progressive joint destruction, resulting in a significant disability rate. The synovium and FLSs, as the primary effector tissues and cells in RA, have long been recognized as critical therapeutic targets for RA treatment. Numerous therapeutic strategies targeting RA FLSs have been investigated.^[^ ^37^ ^]^ In the context of RA drug development, a rigorous evaluation of the efficacy and safety of candidate drugs is essential before they advance to clinical trials. Thus, establishing an appropriate RA synovial model is indispensable. Most prior studies have relied on 2D monolayer cell cultures or animal models. However, 2D cultures are insufficient to fully recapitulate the complex in vivo microenvironment, and animal experiments are limited by ethical considerations and species‐specific differences. Humanized organoids and organ‐on‐a‐chip models show potential for overcoming these limitations and serve as valuable tools for RA drug research.^[^ ^16^, ^38^, ^39^ ^]^ While synovial organoids have been employed in drug and mechanism investigations, single FLSs‐derived organoid models fail to adequately mimic the intricate synovial microenvironment. Moreover, coculturing RA FLSs with other cell types in conventional culture dishes presents challenges in individual cell imaging and the precise targeting of FLSs. Microfluidic chips provide unique advantages in simulating physiological and pathological complexity.^[^ ^24^, ^40^, ^41^ ^]^ Consequently, we chose to develop a synovial model using a microfluidic chip.

For multicellular complex models, determining the optimal proportions of different cell types is a critical issue that requires resolution. In prior studies, the three‐cell spherical coculture model adopted a ratio of RA FLSs/M1 macrophages/HUVECs of 3:1:1, but no detailed justification was provided for this choice.^[^ ^19^ ^]^ Another model utilized a ratio of 1.25:1:2.5, focusing exclusively on the impact of the macrophage density on angiogenesis, and demonstrated that 3 × 10^4^ macrophages were more advantageous for vascular branching and FLSs expansion following the addition of VEGF/bFGF.^[^ ^18^ ^]^ A single‐cell sequencing analysis of 314011 synovial cells revealed an approximate ratio of stromal cells/myeloid cells/endothelial cells of 3:3:1.^[^ ^42^ ^]^ However, accurately replicating the in vivo cellular composition in an in vitro model remains challenging. Given that inflammation constitutes the central pathological process in RA,^[^ ^43^ ^]^ we utilized a comprehensive panel of inflammatory factors as the critical evaluation criteria to determine the optimal ratio among the three cell types, ultimately setting the ratio of RA FLSs/M1 macrophages/HUVECs to 1:1:1. This proportion aligns with the abundant phenotypes of endothelial, fibroblast, and myeloid cells observed in synovial tissue.^[^ ^42^ ^]^ Furthermore, during the dynamic monitoring of the inflammatory profile within our model, the levels of IL‐6, IL‐8, and CXCL‐10 exhibited sustained increases and were significantly different from those in the FM group. Similarly, clinical samples from RA patients (synovial fluid, synovial tissue, and primary synovial cells) presented higher expression levels of IL‐6, IL‐8, and CXCL‐10 than OA samples did. These findings highlight the high clinical relevance of the SOC model we developed and underscore its success and advantages in accurately simulating the pathological state of RA.

FLSs are central to the pathogenesis of RA, as they contribute to the establishment and maintenance of the synovial inflammatory microenvironment. This microenvironment, in turn, perpetuates and exacerbates the inflammatory state, invasiveness, and proliferative capacity of RA FLSs. Therefore, targeting the invasive and proliferative properties of RA FLSs may constitute a promising therapeutic strategy for mitigating disease progression.^[^ ^44^, ^45^, ^46^ ^]^ The development of an in vitro model specifically designed to investigate RA FLSs is crucial for elucidating its pathological mechanisms. In vivo, FLSs interact with various cell types, particularly immune cells and endothelial cells. The interaction between endothelial cells and FLSs promotes inflammation and significantly enhances the proliferative capacity of FLSs,^[^ ^47^ ^]^ as confirmed by our experimental data. Notably, the Ki‐67 expression level of RA FLSs in the FME group was markedly higher compared to those in the FM and F groups (Figure 6B,C). A range of immune cells, including macrophages,^[^ ^48^ ^]^ T cells,^[^ ^49^ ^]^ B cells,^[^ ^50^ ^]^ and neutrophils,^[^ ^51^ ^]^ engage in complex interactions with RA FLSs. However, culturing these immune cells in vitro over extended periods poses technical challenges. Macrophages play a critical role in synovial inflammation, and their interaction with FLSs results in elevated levels of IL‐6 and MMP‐3, findings that are consistent with our observations (Figures 4 and 6B,C). Compared with single‐cell culture systems, multicell coculture models more accurately reflect the in vivo pathological state of RA FLSs, as evidenced by our study. However, the underlying mechanisms by which the concomitant presence of M1 macrophages and endothelial cells exacerbate the pathological phenotype of RA FLSs require further investigation.

This model has distinct advantages in toxicity evaluation. Conventional in vitro cytotoxicity assays are limited to assessing the toxicity of single cell types and cannot evaluate the toxicity toward other cells because of drug delivery or intercellular interactions. This limitation underscores one of the key reasons why in vivo experiments remain essential for drug development. However, animal experimentation is expected to face restrictions beyond 2035.^[^ ^52^ ^]^ Organoids and organ‐on‐a‐chip technologies, as promising alternatives to in vivo studies, are poised to play a more critical role in toxicity assessment.^[^ ^53^, ^54^ ^]^ TP and CEL, the active components of *Tripterygium wilfordii*, have been investigated predominantly for their hepatotoxicity and nephrotoxicity in prior studies,^[^ ^55^, ^56^ ^]^ with little attention given to their synovial toxicity. Previous 3D culture models of synovial tissue have focused primarily on evaluating toxicity at the whole‐spheroid level, and specific analyses of individual cell types are lacking.^[^ ^19^ ^]^ In this study, we systematically evaluated the cytotoxic effects of TP and CEL on three distinct cell types. The results revealed that TP exhibited greater toxicity toward HUVECs, whereas CEL was more toxic to fibroblasts, findings that are consistent with previous reports.^[^ ^57^, ^58^ ^]^ Our findings suggest that the observed pharmacological effects arise from both direct drug diffusion and cell‐cell interactions. However, the precise contribution of each mechanism requires further elucidation. Additionally, more detailed mechanistic studies using this model system remain to be performed.

## 4 Conclusion

In conclusion, we successfully developed a synovium‐on‐a‐chip model that targets RA FLSs by optimizing the Matrigel composition, cell density, and cell ratio. Our model shows robust multicellular interactions, leading to the expression of high levels of IL‐6, IL‐8, and CXCL10, which closely recapitulate the inflammatory profile observed in RA clinical samples. These findings provide a reliable preclinical platform for future RA drug development. In this model, RA FLSs exhibit enhanced invasiveness and proliferation, effectively reproducing key functional characteristics of RA FLSs in vitro. Additionally, we evaluated the effects of two drugs, TP and CEL, using this model and confirmed its ability to accurately reflect both the efficacy and toxicity profiles. Notably, this model enables the monitoring of drug‐induced toxicity in macrophages and endothelial cells following RA FLSs treatment, thereby overcoming the limitations of conventional in vitro models that only assess toxicity in a single cell type. Our model holds significant promise for advancing research into drugs that target FLSs and elucidating the mechanisms underlying intercellular interactions.

## 5 Experimental Section

### Microfluidic Chip Fabrication

The microfluidic chip mold was provided by the collaborative research group at the Chinese Academy of Agricultural Sciences. A mixture of polydimethylsiloxane (PDMS) precursor and curing agent (10:1 w/w, Dow Corning SYLGARD‐184, USA) was carefully prepared and poured into the mold. The mixture underwent vacuum degassing for 30 minutes and was then thermally cured in an oven at 80 °C for 3 hours. After cooling to room temperature, the PDMS slab was carefully detached from the mold and trimmed to the desired dimensions. The PDMS layer was irreversibly bonded to a clean glass substrate following a 90‐second plasma treatment. Finally, the assembled device was sterilized under ultraviolet light for 30 minutes to render it suitable for cell suspension inoculation.

### Primary Cell Extraction and Sample Collection

Synovial tissue and synovial fluid were obtained from patients with RA and OA who underwent joint replacement surgery or joint puncture. Primary FLSs derived from RA and OA patients were isolated from the collected synovial tissue and cultured in Dulbecco's modified Eagle's medium (DMEM; Gibco, USA) supplemented with 15% fetal bovine serum (FBS; Pricella, China). The primary cell phenotype was identified by flow cytometry. The detailed information is provided in the Supporting Information. This study was conducted with the approval of the Ethics Committee of China‐Japan Friendship Hospital (approval number: 2023‐KY‐185). Written informed consent from participants was obtained. Joint sections from CIA rats and K/BxN mice were prepared and provided by the research team.^[^ ^35^, ^59^ ^]^

### 2D Cell Culture

The THP‐1 cells (RRID: CVCL_0006) and HUVECs (RRID: CVCL_2959) used in this study were obtained from the laboratory's cell bank on August 1, 2023, and were confirmed to be correct and free of contamination via Short Tandem Repeat (STR) profiling. HUVECs were maintained in endothelial cell medium (ScienCell, USA). THP‐1 cells were cultured in RPMI‐1640 medium (Gibco, USA) supplemented with 10% FBS (Pricella, China). THP‐1 cells were seeded in culture dishes, incubated for 24 hours, and treated with 100 ng/mL phorbol‐12‐myristate‐13‐acetate (PMA; MedChemExpress, USA) to induce differentiation into MΦ‐type macrophages. Subsequently, induction medium containing 100 ng mL^−1^ lipopolysaccharide (LPS; Sigma‐Aldrich, Germany), 20 ng/mL IFN‐γ (Novoprotein, China), and PMA was prepared in RPMI‐1640 and incubated with the cells for 48 hours to promote their differentiation into M1 macrophage. The phenotypes of the induced cells were identified by IF staining. The detailed information is provided in the Supporting Information.

### 3D Cell Culture

The experiments were performed using RA FLSs, HUVECs and THP‐1 cells at passage numbers between P3 and P5. The cells were cultured in a 2D environment until they reached the appropriate state and density. They were then harvested by centrifugation and resuspended in complete medium consisting of DMEM supplemented with 15% FBS. The resulting cell suspension was mixed with 25% Matrigel (Corning, USA) and adjusted to the optimal cell density prior to seeding into the chip. FLSs were seeded into the central channel, whereas HUVECs and M1 macrophages were seeded into the side channels. All operations involving Matrigel were conducted on ice. These procedures were executed expeditiously to minimize cell stress and prevent premature solidification of the matrix gel. A syringe pump (LONGER, UK) was subsequently utilized to ensure daily fluid exchange after 24 hours.

### IF Staining and Flow Cytometry

The chips were fixed with 4% paraformaldehyde for 10 minutes. The cells were permeabilized with 0.1% Triton X‐100 for 10 minutes, and PBS washes were performed between each step. Nonspecific binding was blocked by incubating the samples with 0.3% bovine serum albumin (BSA) for 1 hour prior to the addition of the primary antibody. The primary antibodies used included those against Vimentin (1:1000, 10366‐1‐AP, Proteintech, China), CD68 (1:1000, 66231‐2‐Ig, Proteintech, China), CD31 (1:1000, 66065‐2‐Ig, Proteintech, China), CDH11 (1:200, A12176, ABclonal, China), iNOS (1:500, 22226‐1‐AP, Proteintech, China) and VE‐Cadherin (1:500, 27956‐1‐AP, Proteintech, China), ICAM‐1 (1:100, 10831‐1‐AP, Proteintech, China). The primary antibodies utilized in this study were incubated with the samples overnight at 4 °C on the microarray. Fluorescent dye‐conjugated secondary antibodies were incubated with the samples for 1 hour at room temperature, followed by nuclear counterstaining with 4′,6‐diamidino‐2‐phenylindole (DAPI; Solarbio, China). Confocal images were acquired using a confocal scanning microscope (Leica, STELLARIS 5, Germany) and analyzed using ImageJ software. For flow cytometry, primary cells were fixed, permeabilized, and subsequently incubated with Alexa Fluor 488‐conjugated anti‐Vimentin (677809; BioLegend, USA) and Elab Fluor 647 Anti‐Human CD68 (E‐AB‐F1299M; Elabscience, China) antibodies. The cells were detected with a Beckman instrument and analyzed using FACSDiva software.

### Cytokine Assay

A LEGENDplex Human Panel (BioLegend, 12547, USA) was utilized to detect cytokines in the supernatants of the synovium‐on‐a‐chip using a flow cytometer (Beckman, CytoFLEX LX, USA). Other indicators were quantified in the collected synovium‐on‐a‐chip supernatants using commercially available ELISA kits according to the manufacturer's instructions. The levels of IL‐6, IL‐8, CXCL10, MMP‐1, and MMP‐3 were examined using ELISA kits (E‐OSEL‐H0001, E‐OSEL‐H0014, E‐EL‐H0050, E‐EL‐H6073, and E‐EL‐H1446; Elabscience, China) according to the manufacturer's instructions.

### mIHC and IHC

Synovial tissues obtained from RA and OA patients were sectioned. The tissue sections were deparaffinized with xylene, rehydrated through a graded ethanol series, and subjected to antigen retrieval by an incubation with 10% goat serum. For mIHC, the tissue sections were stained using the corresponding kits (Immunoway, RS0035, USA). The staining procedure, including the primary antibody incubation, secondary antibody incubation, TSA signal amplification, repeated staining cycles, and nuclear staining, was performed stepwise in strict accordance with the manufacturer's instructions. Confocal images were acquired using a confocal scanning microscope. The primary antibodies used for mIHC were identical to those used for the IF analysis. For IHC, the tissue sections were subsequently incubated with the primary antibodies overnight at 4 °C, followed by an incubation with the appropriate secondary antibody. The primary antibodies used were CD68 (1:1000, 66231‐2‐Ig, Proteintech, China), CD34 (1:1000, 14486‐1‐AP, Proteintech, China), MMP‐3 (1:200, 17873‐1‐AP, Proteintech, China), IL‐6 (1:200, A21264, ABclonal, China), IL‐8 (1:200, 27095‐1‐AP, Proteintech, China) and CXCL10 (1:500, 10937‐1‐AP, Proteintech, China) antibodies. The sections were then processed with a DAB detection kit (PV‐6000D; ZSGB‐BIO, China) for chromogenic reactions.

### Cell Viability Assay

The viability of cells in 3D culture was evaluated using a Calcein/PI Cell Viability/Cytotoxicity Assay Kit (Beyotime, China) according to the manufacturer's instructions. The staining solution was diluted 1000‐fold with PBS and subsequently injected into the medium channels of the chips. The samples were incubated for 30 minutes at 37 °C. After the incubation, the cells were imaged using a confocal scanning microscope. For each sample, images were acquired from three distinct areas. Cell viability was quantified by counting red (dead) and green (viable) cells using ImageJ software. The viability of cells in 2D culture was evaluated using Cell‐Counting Kit‐8 (CCK‐8; MA0218; MeilunBio, China). A 10% CCK‐8 solution was added to each 2D well, and the plate was maintained in an incubator at 37 °C for 2 h. The absorbance at 450 nm was measured with a microplate reader (Thermo Fisher, China).

### Statistical Analysis

Statistical analyses were conducted on the data obtained from three independent experiments. The data are presented as the mean ± standard deviation (SD). Independent samples *t*‐test for two groups and one‐way analysis of variance (ANOVA) for multiple groups were performed using Origin software to evaluate the statistical significance of the results. Tukey's or Dunnett's honestly significant difference (HSD) test was employed for post hoc comparisons between groups. Sample size was no less than 3 for each statistical analysis. *P *< 0.05 was considered statistically significant.

## Conflict of Interest

The authors declare no conflict of interest.

## Author Contributions

W.D. wrote the original draft, performed the data curation, and conceptualized the idea for the study. Y.J. performed validation and developed the software. T.D. performed the supervision. J.G. performed the formal analysis. B.W., Q.Z., and P.W. helped in acquiring resources. N.X. and C.X. performed the supervision and project administration. All authors contributed to the article and approved the submitted version.

## Supporting information



Supporting Information

## Data Availability

Data sharing is not applicable to this article as no new data were created or analyzed in this study.
