# Supplementary material for: Synovium‐On‐A‐Chip: Simulating the Microenvironment of the Rheumatoid Arthritis Synovium via Multicell Interactions to Target Fibroblast‐Like Synoviocytes
Source: Adv Sci (Weinh). 2025 Sep 23;12(46):e11945. doi: 10.1002/advs.202511945 (PMC12697785; doi:10.1002/advs.202511945)
Supplement: Supplementary file 1 — Supporting Information [file ADVS-12-e11945-s001.docx]

**Supporting Information**

**Title: Synovium-on-a-chip: Simulating the Microenvironment of the Rheumatoid Arthritis Synovium via Multicell Interactions to Target Fibroblast-Like Synoviocytes**

*Wenya Diao, Yi Jiao, Tingting Deng, Jienan Gu, Bailiang Wang, Qidong Zhang, Peilong Wang, Ning Xu*, Cheng Xiao**

W. Diao, Y. Jiao, J. Gu, C. Xiao

China**–**Japan Friendship Clinical Medical College, Beijing University of Chinese Medicine, Beijing 100029, China

Email: [xuning@caas.cn](mailto:xuning@caas.cn); [xc2002812@126.com](mailto:xc2002812@126.com)

W. Diao, Y. Jiao, T. Deng, J. Gu, C. Xiao

Institute of Clinical Medical Sciences, China**–**Japan Friendship Hospital, Beijing 100029, China

B. Wang, Q. Zhang

Department of Orthopedics, China**–**Japan Friendship Hospital, Beijing 100029, China

P. Wang, N. Xu

Institute of Quality Standard and Testing Technology for Agro–Products, Chinese Academy of Agricultural Sciences, Beijing 100081, China

**Supplementary Figures**


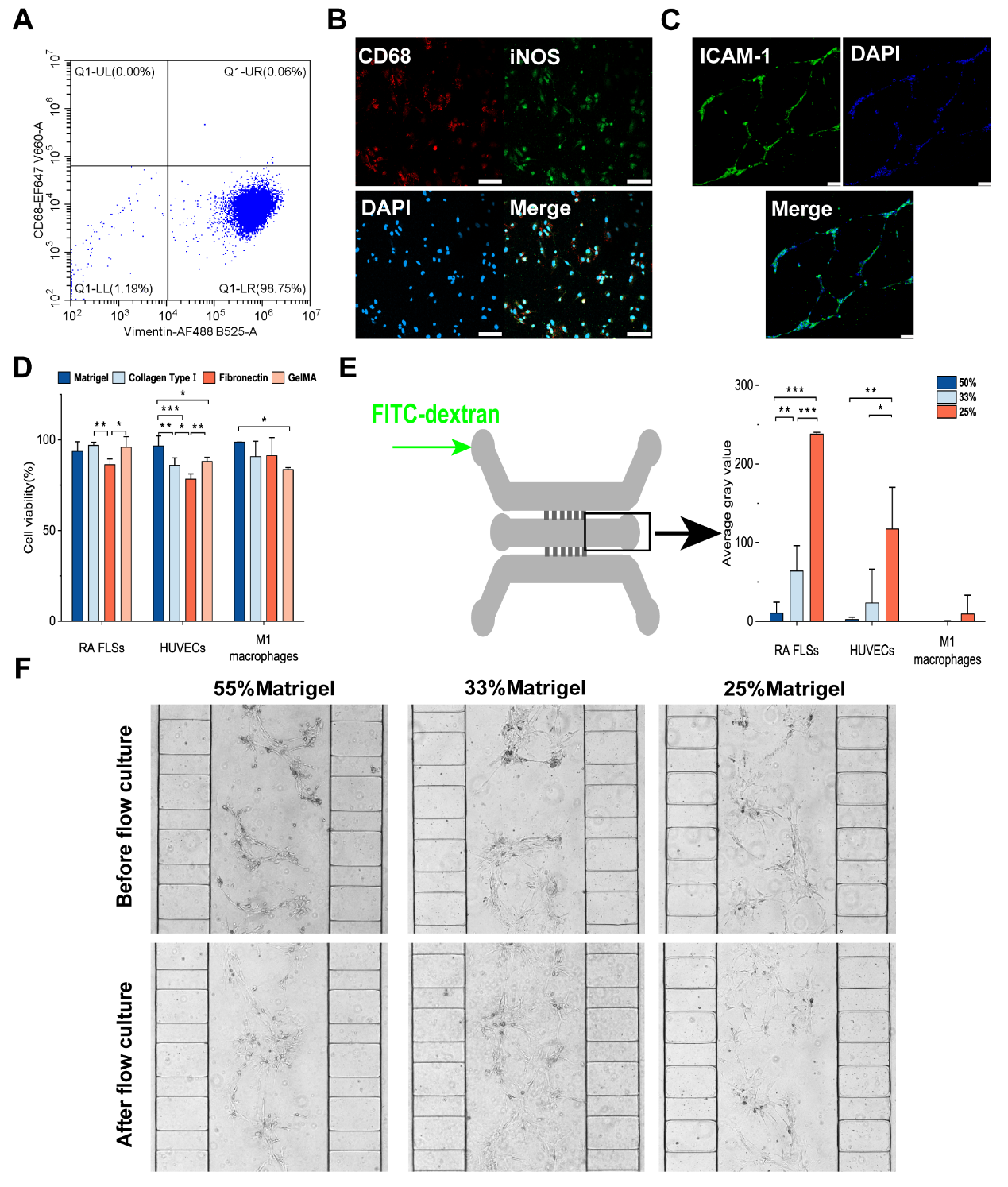


**Figure S1. Characterization of cell phenotypes and optimization of cell culture matrices.**

(A) Primary fibroblasts isolated from synovial tissue were identified as Vimentin^+^CD68^-^ by a flow cytometry analysis. (B) M1 macrophages derived from THP-1 cells were confirmed to be CD68^+^iNOS^+^ through immunofluorescence staining. Scale bars, 100 μm. (C) HUVECs in 3D culture were confirmed to be ICAM-1^+^ through immunofluorescence staining. Scale bars, 100 μm. (D) The survival rates of RA FLSs, HUVECs and M1 macrophages were evaluated in four different culture matrices: Matrigel, type I collagen from rat tail (collagen type I), fibronectin, and gelatin methacryloyl (GelMA). (E) The mean gray value of RA FLSs, HUVECs, and M1 macrophages were measured after the perfusion of FITC-dextran through the side channel in varying concentrations of Matrigel (50%, 33%, and 25%). (F) Microscopic bright-field images of RA FLSs before and after flow culture in varying concentrations of Matrigel (50%, 33%, and 25%). *n*=3 per group. Data are presented as the mean ± SD. Statistical analysis: one-way ANOVA with Tukey’s test. **p* < 0.05, ***p* < 0.01, and ****p* < 0.001.


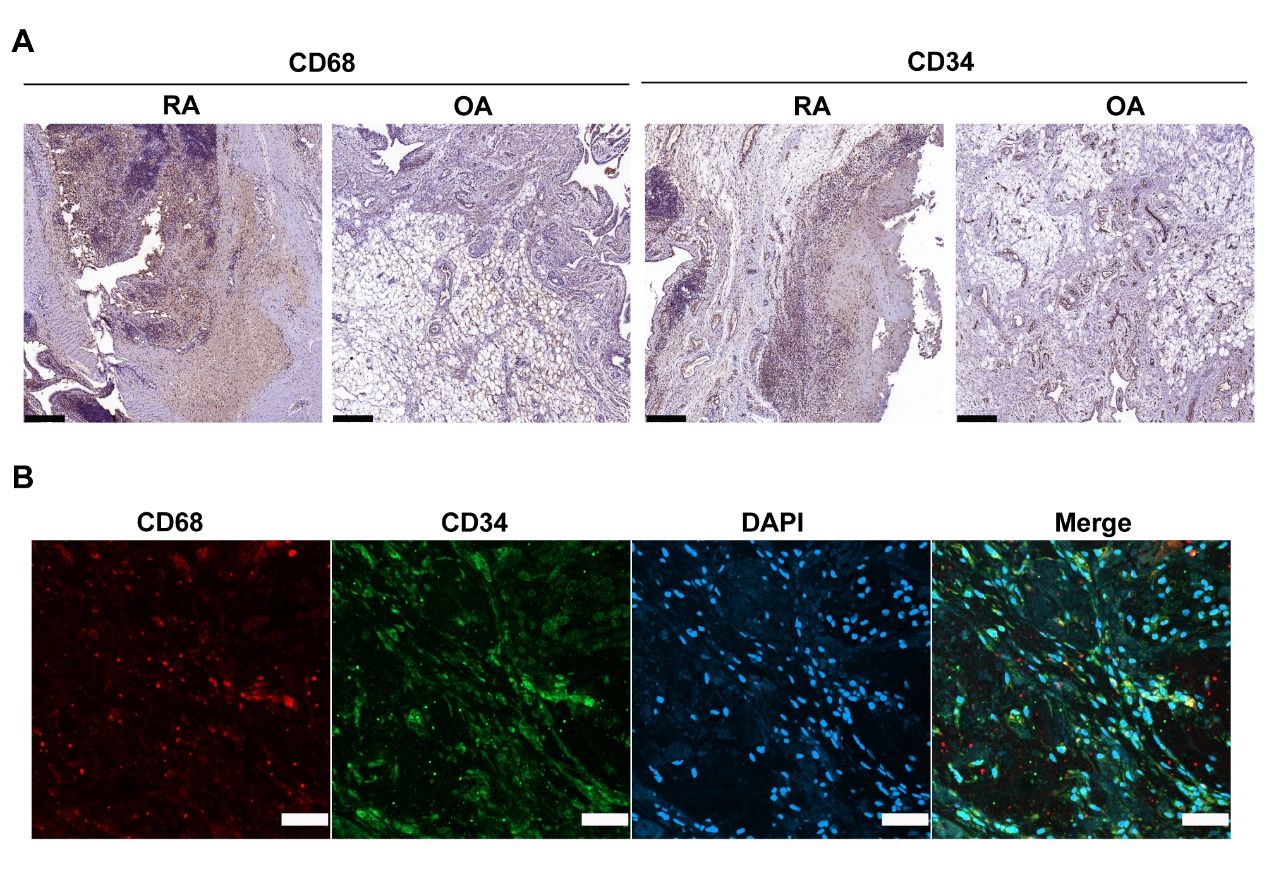


**Figure S2. Expression of CD68 and CD34 in synovial tissue.**

(A) Representative images of immunohistochemical staining for CD68 and CD34 in synovial tissues from RA and OA patients. Scale bars: 500 μm.

(B) Representative images of immunofluorescence staining for CD68 and CD34 in synovial tissue from RA patient. Scale bars: 50 μm.


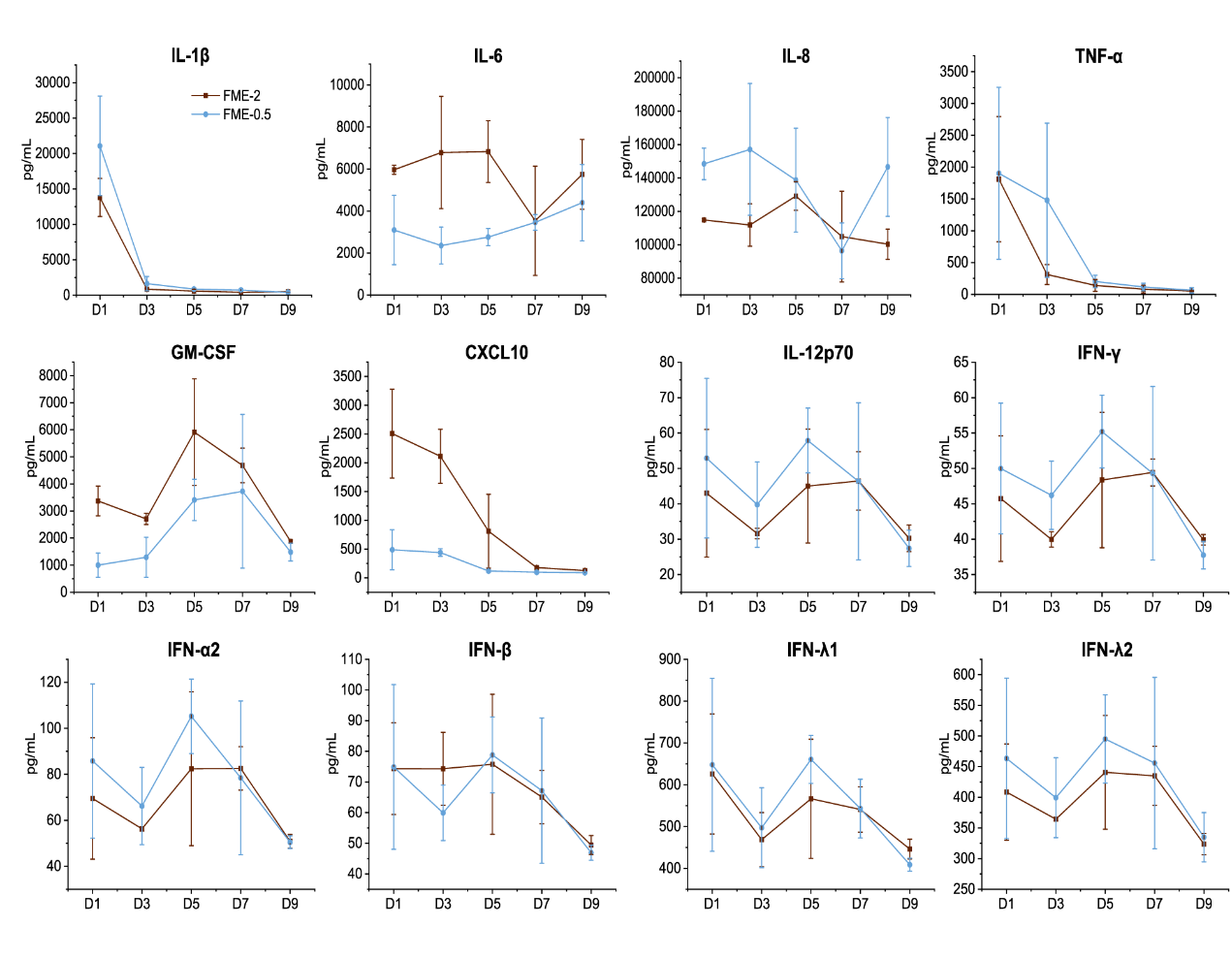


**Figure S3. Expression levels of inflammatory factors on different days (days 1–9) in the FME-2 and FME-0.5 groups.** *n*=3 per group.


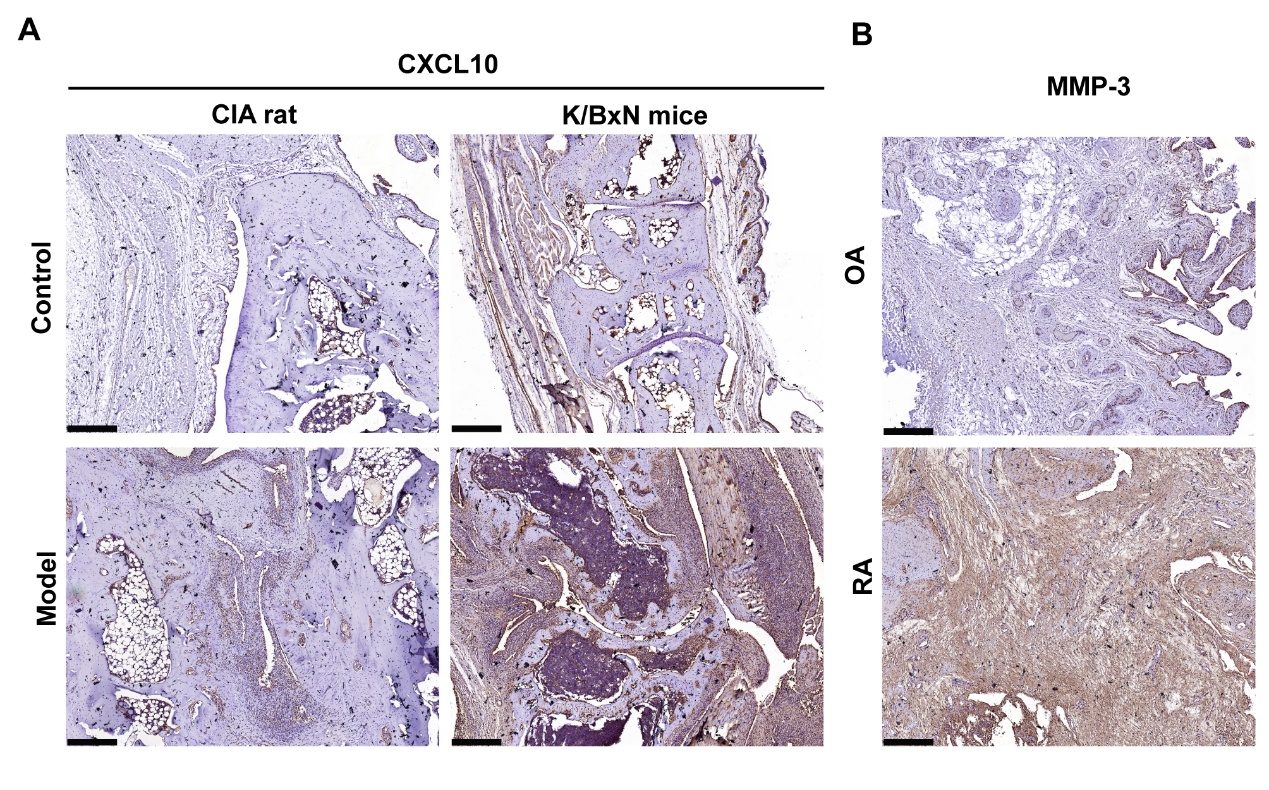


**Figure S4. Expression of CXCL10 within the joints and MMP-3 in synovial tissue.**

(A) Representative images of immunohistochemical staining for CXCL10 in the joints of the collagen-induced arthritis (CIA) rats model and K/BxN mouse joints. Scale bars: 500 μm.

(B) Representative images of immunohistochemical staining for MMP-3 in synovial tissues from RA and OA patients. Scale bars: 500 μm.


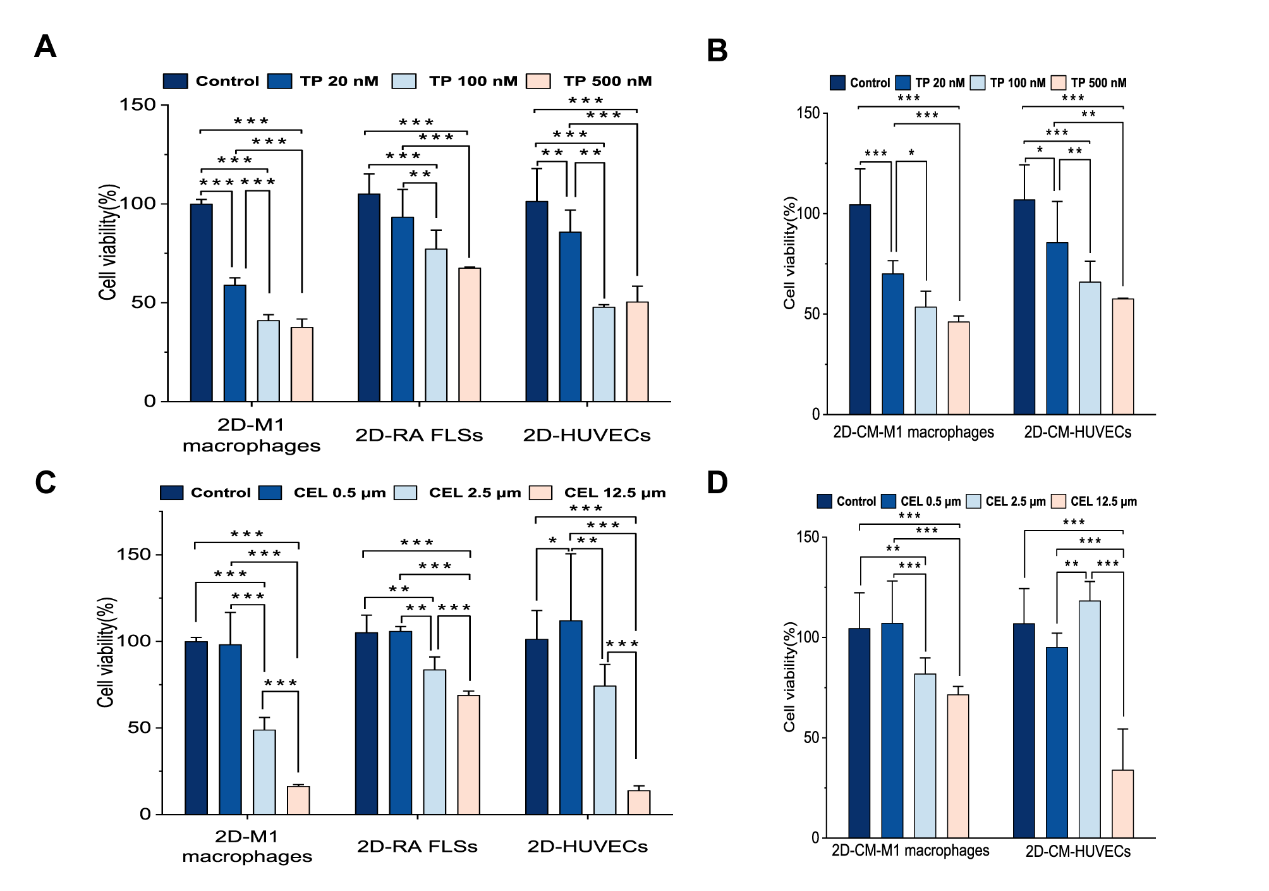


**Figure S5. Evaluation of the toxicity of TP and CEL in two-dimensional (2D) cell culture.**

(A) Survival rates of 2D-M1 macrophages, 2D-RA FLSs, and 2D-HUVECs after TP administration. (B) Survival rates of M1 macrophages and HUVECs cultured in the supernatant of 2D-FLSs (2D-CM) after TP administration. (C) Survival rates of 2D-M1 macrophages, 2D-RA FLSs, and 2D-HUVECs after CEL administration. (D) Survival rates of M1 macrophages and HUVECs cultured in the 2D-CM after CEL administration. *n*=3 per group. Data are presented as the mean ± SD. Statistical analysis: one-way ANOVA with Tukey’s test. **p* < 0.05, ***p* < 0.01, and ****p* < 0.001.

**Supplementary Table**

**Supplementary Table 1: Characteristics of the patients included in this study.** This study was conducted with the approval of the Ethics Committee of the China-Japan Friendship Hospital (approval number: 2023-KY-185). Written informed consent from participants was obtained. **(**ESR=erythrocyte sedimentation rate, F=female, M=male.)

| RA | | | | OA | | | |
| --- | --- | --- | --- | --- | --- | --- | --- |
| ID | Sex | Age(years) | ESR(mm/h) | ID | Sex | Age(years) | ESR(mm/h) |
| RA-01 | F | 53 | 57 | OA-01 | F | 68 | 8 |
| RA-02 | F | 64 | 11 | OA-02 | F | 65 | 23 |
| RA-03 | F | 66 | 34 | OA-03 | F | 79 | 5 |
| RA-04 | F | 55 | 82 | OA-04 | F | 55 | 13 |
| RA-05 | F | 69 | 12 | OA-05 | F | 63 | 7 |
| RA-06 | F | 37 | 13 | OA-06 | F | 77 | 21 |
| RA-07 | F | 62 | 11 | OA-07 | F | 66 | 5 |
| RA-08 | F | 58 | 22 | OA-08 | F | 69 | 7 |
| RA-09 | F | 69 | 90 | OA-09 | F | 74 | 23 |
| RA-10 | F | 55 | 19 | OA-10 | F | 72 | 21 |
| RA-11 | F | 48 | 25 | OA-11 | F | 74 | 6 |
| RA-12 | F | 69 | 26 | OA-12 | F | 63 | 12 |
| RA-13 | F | 49 | 8 | OA-13 | M | 70 | 6 |
| RA-14 | F | 53 | 13 | OA-14 | F | 78 | 25 |
| RA-15 | F | 63 | 14 | OA-15 | F | 67 | 7 |
| RA-16 | F | 64 | 13 | OA-16 | F | 74 | 7 |
| RA-17 | F | 48 | 49 | OA-17 | F | 68 | 5 |
| RA-18 | F | 56 | 99 | OA-18 | F | 70 | 13 |
| RA-19 | F | 69 | 23 | OA-19 | F | 79 | 12 |
| RA-20 | F | 48 | 2 | OA-20 | F | 64 | 15 |
| RA-21 | F | 53 | 7 | OA-21 | M | 62 | 2 |
| RA-22 | F | 61 | 42 | OA-22 | F | 64 | 7 |
| RA-23 | F | 53 | 53 | OA-23 | F | 69 | 28 |
| RA-24 | F | 53 | 15 | OA-24 | F | 67 | 14 |
